# Importance of Diversity in the Oral Microbiota including *Candida* Species Revealed by High-Throughput Technologies

**DOI:** 10.1155/2014/454391

**Published:** 2014-04-22

**Authors:** Tamaki Cho, Jun-ichi Nagao, Rieko Imayoshi, Yoshihiko Tanaka

**Affiliations:** Section of Infection Biology, Department of Functional Bioscience, Fukuoka Dental College, 2-15-1 Tamura, Sawara-ku, Fukuoka 814-0193, Japan

## Abstract

Taking advantage of high-throughput technologies, deep sequencing of the human microbiome has revealed commensal bacteria independent of the ability to culture them. The composition of the commensal microbiome is dependent on bacterial diversity and the state of the host regulated by the immune system. *Candida* species are well known as components of the commensal oral microbiota. *Candida* species frequently colonize and develop biofilms on medical devices like dentures and catheters. Therefore, *Candida* biofilm on dentures leads to a decrease in the bacterial diversity and then to a change in the composition of the oral microbiota. A disturbance in the balance between commensal bacteria and the host immune system results in a switch from a healthy state to a diseased state even in the limited oral niche.

## 1. Introduction

**Figure 1:**
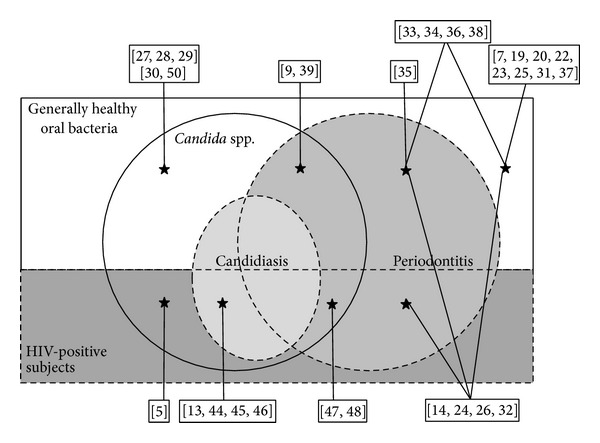
The microbiota and diseases described in the literature are cited in this review. The white background represents oral bacteria from generally healthy subjects. The dark grey background represents oral bacteria from HIV-positive subjects. The circle of* Candida* spp. represents detection of* Candida* spp. from oral bacteria in subjects. The light gray circle of candidiasis represents subjects with candidiasis. The gray circle of periodontitis represents subjects with periodontitis. Numbers in squares represent number of references cited in the text.

The progression of a global aging population has been accompanied by severe problems in oral health because aging induces risk factors like tooth loss, wearing dentures, senescence of tissues, and systemic diseases (diabetes, malignancies, implantations of tissues, etc.), which may disturb the oral homeostasis [1–10].* Candida* species, especially* Candida albicans,* is a normal component of the human flora. However,* C. albicans* causes oral candidiasis in immunocompromised hosts [11–13]. On the other hand, periodontitis is also induced by a change in the balance between oral bacteria and the host defense mechanisms [14–16]. The role of* Candida* species in the oral microbiota has not been well studied. Here, as shown in Figure 1, the presence of* Candida* species and the pathogens of periodontitis in generally healthy subjects is described. Moreover, the microbiology of periodontitis including* Candida* species in HIV infected patients is described.

## 2. Microbiome and Mycobiome in the Oral Cavity

Normal control samples are necessary to determine whether there are associations between changes in the microbiome and health/disease and also to yield insights into the role of the endogenous flora in health and disease [17–19]. The NIH Human Microbiome project was funded to take advantage of high-throughput technologies using metagenomic sequencing approaches to characterize the human microbiome on multiple body sites (the gastrointestinal (GI) tract, the mouth, the vagina, the skin, and the nasal cavity) from healthy volunteers [20–22]. The distribution by body site of bacteria in the NIH project [20] showed that the distribution on the oral sites (buccal mucosa, hard palate, keratinized gingiva, palatine tonsils, saliva, sub- and supragingival plaques, throat, and tongue dorsum) was 26% of the total (*n* = 250).

The oral cavity is comprised of many microbial habitats, such as teeth, gingival sulcus, attached gingiva, tongue, cheek, lip, and hard and soft palates. Mager et al. [23] analyzed the proportion of 40 bacterial species on different intraoral surfaces of 225 healthy subjects using checkerboard DNA-DNA hybridization. The proportions of bacterial species differed markedly on different intraoral surfaces. For example,* Actinomyces* spp. colonized teeth at higher proportions than soft tissues. The microbiota of saliva was most similar to that of the dorsal and lateral surfaces of the tongue.* Prevotella melaninogenica*,* Veillonella parvula,* and* Streptococcus mitis* were found in higher proportions on soft tissue surfaces. Paster et al. [24] estimated 500 species in subgingival plaque of periodontally healthy subjects (*n* = 2), periodontitis subjects (*n* = 9), HIV periodontitis patients (*n* = 2), and acute necrotizing ulcerative gingivitis subjects (*n* = 4) using culture-independent molecular techniques (16S rRNA cloning). Also they reported that known putative periodontal pathogens such as* Porphyromonas gingivalis* and* Bacteroides forsythus *(*Tannerella forsythia*) were identified from multiple subjects, but as minor components of the plaque as seen in cultivable studies. Aas et al. [25] defined the human microflora of the healthy oral cavity. The breadth of bacterial diversity in the oral cavity (dorsum of the tongue, lateral sides of the tongue, buccal fold, hard and soft palates, labial gingiva, tonsils, and supra- and subgingival plaques from tooth surfaces) in 5 healthy subjects was analyzed using 16S rRNA identification. It was reported that species common to all oral sites belonged to the genera* Gemella*,* Granulicatella*,* Streptococcus*, and* Veillonella. *Moreover, they identified 13 new phylotypes. A distinctive predominant bacterial flora of the healthy oral cavity was highly diverse and site and subject specific. As much as 60% of the species detected have not been cultivated.

Dewhirst et al. [26] established the Human Oral Microbiome Database (HOMD) based on analysis of 16S rRNA gene clone libraries created in published and unpublished studies from oral health and disease statuses including periodontitis, HIV periodontitis, acute necrotizing ulcerative gingivitis, caries, endodontic infections, and NOMA. The analysis identified 1,179 taxa, of which 24% were named, 8% were cultivated but unnamed, and 68% were uncultivated phylotypes.

Although the oral microbiome has been analyzed in detail, Ghannoum et al. [27] first characterized the fungi present in the oral cavity of 20 healthy subjects using a novel multitag pyrosequencing approach with the pan-fungal internal transcribed spacer (ITS) primers. Sampling was obtained by oral rinses with phosphate buffered saline. The collection of organisms from the dorsum of the tongue and the oral mucosal environment was possible by rinsing.* Candida *species were isolated from 75% of all study participants (21–60 years of age). Although environmental fungi such as* Cladosporium*,* Aureobasidium*, Saccharomycetales,* Aspergillus*,* Fusarium*, and* Cryptococcus* were unexpected in the oral cavity of healthy individuals, they were detected in the oral wash samples. These could simply be spores inhaled from the air or material ingested with food.

Denture wearers can present an increase in the number of* Candida* species cells [28, 29]. Kraneveld et al. [30] studied the relationship between the* Candida* load and the bacterial microbiome profiles of saliva of elderly Dutch adults. Unstimulated saliva was collected for 5 minutes from 82 adults (58–80 years of age). The subjects included a dentate with or without partial prosthesis and an edentate with full upper and lower dentures. Ninety-seven % of the subjects were positive for the* Candida*-specific internal transcribed spacer (ITS) gene. There was a negative correlation between the* Candida* load and the bacterial profiles of saliva. With increased* Candida* load the diversity of the salivary microbiome decreased and the composition changed towards dominance by streptococci and lactobacilli and the disappearance of genera within Fusobacteria and Bacteroidia classes. Therefore, decreased bacterial diversity could be associated with a disbalanced community.

## 3. Oral Microbiota including **Candida** Species in Periodontitis Patients and HIV Infected Patients 

Simon-Soro et al. [31] reported an interesting study regarding sampling sites from oral cavity. They determined the bacterial diversity of different oral microniches of 2 healthy volunteers. Samples were taken from each vestibular and lingual surface of the individual's teeth and gingival sulcus, as well as samples from the tongue dorsum and nonstimulated and stimulated saliva. Interestingly, streptococci are found at higher proportions on the vestibular sulcus compared with lingual sulcus, whereas the highest proportions of obligate anaerobes like* Fusobacterium* were at lingual sites. Nonstimulated saliva samples were not representative of supra- and subgingival plaque. The results from saliva sample accord with the results of Mager et al. [23].

Liu et al. [32] analyzed the oral microbiome in supra- and subgingival plaques of 3 healthy individuals and 2 periodontitis patients who were in good general health. In healthy samples, Gram-positive genera* Streptococcus*,* Actinomyces*, and* Granulicatella* were significantly enriched; however,* Fusobacterium* and* Porphyromonas* were not predominantly in the periodontal disease samples. A microbiota of one of 3 healthy subjects resembled the diseased samples. The control subjects exhibited mild bleeding at probing time, but no attachment loss. Therefore, the control subject could be in initial periodontal disease. The authors suggested that it is necessary to analyze carefully the clinical data collected during sampling. Also the observation may indicate that the microbiota shifts into a disease state before the full clinical symptoms of the disease are apparent. Moreover, Kumar et al. [33] assessed subgingival plaque samples by cloning and sequencing 16S rRNA genes and proved that changes in periodontal health status were associated with bacterial community shifts. Therefore, we understand that the bacterial community is changed from periodontal health to periodontal disease. However, the relative contributions of the host immune system and the shift of the bacterial community in clinically healthy states as suggested by Liu et al. [32] are not well known. Darveau [34] also pointed out the importance of investigating the mechanisms that maintain the stability of, or induce changes in, the microbial composition. Faveri et al. [35] researched the microbiological diversity in subgingival plaque samples from subjects with early onset periodontitis using 16S rRNA clonal analysis.* Selenomonas* and* Streptococcus* accounted for 50% of the clone libraries from the plaque. Therefore, the results showed microbiological diversity in the plaque was altered and decreased. Divaris et al. [36] and Wade [37] insisted that an understanding of host-microbiome interactions is highly important because periodontitis is not an infectious disease in the classical sense, but results from a complex interaction between the commensal microbiota, host susceptibility, and environmental factors. For example, Ge et al. [38] studied factors including smoking, race, and dental caries that may influence subgingival bacterial diversity. Periodontal disease and these influences were found to be independent.

McManus et al. [39] investigated whether clades (a group composed of one ancestor and its descendants) of* C. albicans* isolated from periodontal pockets and periodontal healthy subgingival sites in patients with untreated periodontitis were associated with periodontitis. Specific clonal groups were analyzed by the multilocus sequence typing (MLST) method [40–42].* C. albicans* was isolated from samples of periodontal pockets, healthy subgingival sites, and oral rinses from untreated periodontal subjects. Thirty-one isolates from periodontal pockets belonged to 19 sequence typings, with 11 isolates belonging to MLST clade 1. Sixteen* C. albicans* isolates from oral rinses of healthy subjects belonged to 16 sequence types, with 4 isolates belonging to clade 1. The distributions of sequence types between healthy subjects and periodontitis patients were significantly different. According to other reports, virulence factors and sites of isolation are associated with clade specificity [42, 43]. However, it is not clear if the presence of* C. albicans* in the periodontal pockets contributes to the progression of periodontitis.

The rate of oral* C. albicans* carriage in HIV-positive subjects is higher than in control subjects, and patients with CD4+-cell counts of 200–400/microliter had a significantly higher level of yeast carriage [44]. The samples were collected from the buccal mucosa, the floor of the mouth, and the dorsal surface using a sterile cotton swab.* C. albicans* was the most prevalent agent of oral candidiasis in HIV-positive subjects from resource-rich countries [45]. Oral* Candida* colonization was not affected with antiretroviral therapy in HIV-positive patients [46]. Moreover,* C. albicans* was recovered most often from oral and subgingival samples in HIV-positive subjects with advanced periodontal disease [47]. There is an increase in the prevalence of* Candida* species in the subgingival plaque of HIV-seropositive patients compared to immunocompetent subjects [48]. Aas et al. [49] compared the predominant bacterial and fungal species associated with gingivitis (*n* = 5), periodontitis (*n* = 8), and linear gingival erythema (LEG [50], *n* = 1) in 14 HIV-positive subjects. Samples were isolated with cotton rolls from supra- and subgingival plaque. The classical periodontal pathogens,* Treponema denticola*,* P. gingivalis,* and* T. forsythia,* were not detected. The predominant bacterial species were potential opportunistic pathogens,* Gemella*,* Dialister*,* Streptococcus, *and* Veillonella*. In 2 of 4 subjects with low viral loads, high CD4 levels, and periodontitis,* C. albicans* was predominant and* S. cerevisiae* was only a minor component. However,* S. cerevisiae* (not* C. albicans*) was the only fungal species detected from the LGE subject and 2 of 4 severe subjects with high viral loads, low CD4 levels, and periodontitis. These data were indicative of opportunistic infection in a highly susceptible immunocompromised host.

## 4. Conclusion

**Figure 2:**
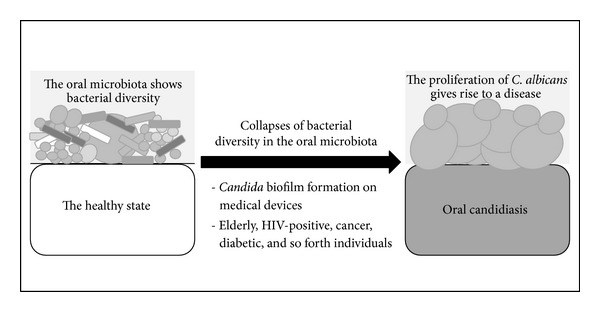
Illustration of the shift in the commensal microbiota in the healthy state relative to the disease state (see Section 4).

The oral healthy state is in optimum balance between the oral microbiota and the host immune system (Figure 2). The healthy state maintains the diversity of the oral microbiota. A reduction in diversity such as* Candida* biofilm formation on dentures may respond to the host immune system, resulting in the shift into a disease state though the symptoms of the disease are not always apparent.
